# Optical coherence tomography angiography findings of choroidal neovascularization secondary to decalcified choroidal osteoma treated with intravitreal aflibercept: A case report

**DOI:** 10.1097/MD.0000000000021472

**Published:** 2020-07-24

**Authors:** Bangtao Yao, Fei Wang, Xiaogui Zhao, Bei Wang, Gang Liu, Yuhua Ding

**Affiliations:** a Department of Ophthalmology, Lishui District People's Hospital, Lishui Branch of Southeast University Affiliated Zhongda Hospital; b Department of Ophthalmology, Jiangsu Province Hospital, The First Affiliated Hospital of Nanjing Medical University, Nanjing, Jiangsu Province, China.

**Keywords:** best-corrected visual acuity, choroidal neovascularization, choroidal osteoma, intravitreal aflibercept, optical coherence tomography angiography

## Abstract

**Introduction:**

Choroidal osteoma (CO) is a rare benign ocular tumor characterized by ossifying choroid lesions. To the best of our knowledge, this is the first detailed report to describe the optical coherence tomography angiography (OCTA) findings of choroidal neovascularization (CNV) complicated by a rare decalcified CO following intravitreal aflibercept injection.

**Patient concerns:**

A 26-year-old woman presented with a spontaneous but painless reduction in visual acuity in her left eye that had commenced 5 days prior.

**Diagnosis:**

Clinical manifestations and multimodal imaging, including OCTA, spectral-domain optical coherence tomography, fundus fluorescein angiography and indocyanine green angiography, indicated decalcified CO with CNV.

**Interventions:**

After receiving an appropriately detailed explanation of the procedure, the patient was treated via intravitreal aflibercept(2.0 mg) injection once.

**Outcomes:**

One month after the therapy, OCTA revealed that the flow areas of CNV were narrowed, the best-corrected visual acuity was improved to 20/30, however, 2 months follow-up OCTA revealed that the CNV reoccurred, the best-corrected visual acuity was dropped to 20/50.

**Conclusion:**

Intravitreal aflibercept injection was an effective treatment for CO with CNV, but the effects may not last long. The OCTA findings provide a better appreciation of evaluating the effects of intravitreal aflibercept injection for CO complicating CNV.

## 1 Introduction

Choroidal osteoma (CO) is a rare benign ocular tumor characterized by an ossifying lesion within the choroid that is well defined, located in the juxtapapillary or macular region, and tends to occur unilaterally.^[1]^ Reports have suggested that the CO-associated reductions in vision are typically due to choroidal neovascularization (CNV) or subretinal fluid (SRF).^[2]^

Optical coherence tomography angiography (OCTA) is a noninvasive imaging modality for depth-resolved visualization of retinal vasculature, which provides a high-resolution image of the microvasculature of the different layers in the retina.^[3]^ It can detect the CNV and observe the morphology and flow areas of CNV without obscured details due to dye leakage in CO.^[4]^

Aflibercept (Eylea; Regeneron Pharmaceuticals, Inc, Tarrytown, NY) has been introduced as a new anti-vascular endothelial growth factor (VEGF) agent, has high binding affinity for all VEGF isoforms and exerts strong antiangiogenic effects. Aflibercept has been proven safe and effective in neovascularisation diseases, such as diabetic retinopathy and age-related macular degeneration.^[5]^ However, the use of aflibercept for CNV secondary to CO has been rarely reported. To the best of our knowledge, this is the first detailed report to describe the OCTA findings of CNV complicated by a rare decalcified CO following intravitreal aflibercept injection.

## 2 Case presentation

**Figure 1:**
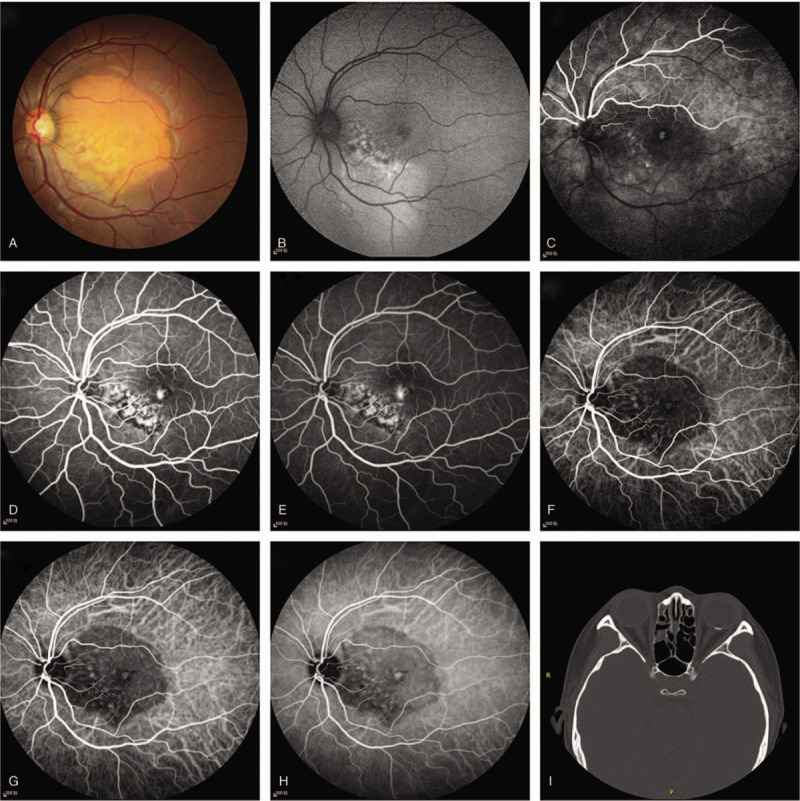
FFA and ICGA images of the patient. (A) Fundus examination revealed a yellow-orange lesion in the macular region of her left eye. (B) Fundus autofluorescence revealed patchy hyper-fluorescence appearance underneath the macula and hypo-fluorescence in the fovea. (C, D, E) FFA revealed patchy hyper-fluorescence in the fovea with the evidence of leakage in the late phase. (F, G, H) ICGA demonstrated hypo-fluorescence corresponding to the lesion, patchy hyper-fluorescence with leakage in the late phase. (**i**) Computed tomography revealed a hyperdense plaque predominantly located at the posterior pole of the left eye. ICGA = indocyanine green angiography.

**Figure 2:**
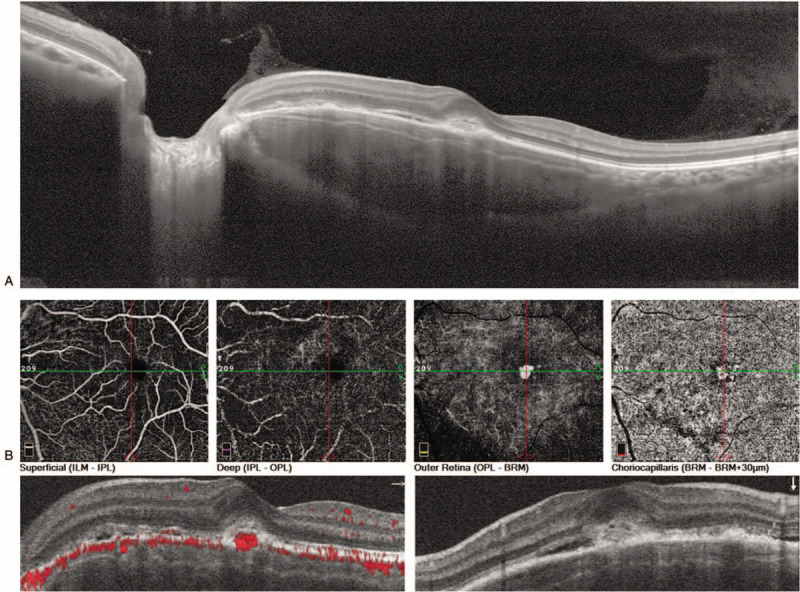
SD-OCT and OCTA images the patient before treatment. (A) The SD-OCT revealed an elevated lesion in the macular region with a lump-like hyper-reflection indicating CNV associated with subretinal fluid (SRF) in the left eye, disorganization of out retina and Bruch membrane was also observed. (B) OCTA and B-scan demonstrated the CNV broke through choriocapillaris layer into the outer retina layer, the superficial retina layer and deep retina layer were normal. SD-OCT = spectral domain optical coherence tomography. SRF = subretinal fluid.

**Figure 3:**
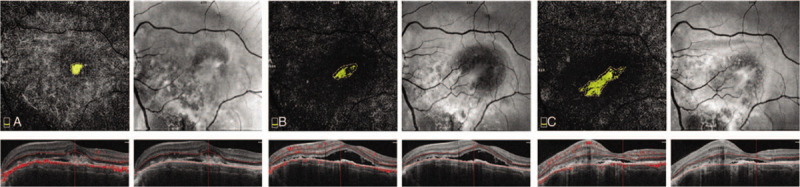
OCTA images of out retina layer with associated B-scans of the patient. (A) before treatment, the CNV and SRF were observed, the flow areas were 0.197 mm^2^. (B) One month after the intravitreal aflibercept injection, OCTA and B-scan revealed that the SRF was increased but the flow areas of CNV were narrowed to 0.191 mm^2^. (**C**) Two months follow-up OCTA revealed that the CNV reoccurred, the flow areas of CNV were expanded to 0.666 mm^2^. SRF = subretinal fluid.

A 26-year-old woman presented with a spontaneous but painless reduction in visual acuity in her left eye that had commenced 5 days prior. Her systemic and ophthalmic histories were unremarkable. The best-corrected visual acuity (BCVA) was 20/20 in the right eye and 20/50 in the left eye. The anterior segment was clear, and the intraocular pressure was normal. Fundus examination revealed a yellow-orange lesion including yellow-white decalcified lesions within its center in the macular region of her left eye (Fig. 1(A)). Fundus autofluorescence in the left eye revealed patchy and mottled hyper-fluorescence appearance underneath the macula and hypo-fluorescence in the fovea (Fig. 1(B)). Fundus fluorescein angiography revealed patchy hyper-fluorescence in the fovea with the evidence of leakage in the late phase and mottled hyper-fluorescence in the inferior-nasal area of the lesion in the early phase, which stained during the sequence (Fig. 1(C) (D) (E)). Indocyanine green angiography demonstrated hypo-fluorescence corresponding to the lesion, with patchy hyper-fluorescence in the fovea in the early phase, while with leakage in the late phase, and it also demonstrated mottled hyper-fluorescence in the inferior-nasal area of the lesion, which degraded in the late phase (Fig. 1(F) (G) (H)). The spectral domain optical coherence tomography revealed an elevated lesion in the macular region with a lump-like hyper-reflection indicating CNV associated with subretinal fluid (SRF) in the left eye, disorganization of out retina and Bruch membrane with relative preservation of ellipsoid zone were also observed (Fig. 2(A)). OCTA and B-scan demonstrated the CNV broke through choriocapillaris layer into the outer retina layer, the superficial retina layer and deep retina layer were normal (Fig. 2(B)), the SRF was observed, the flow areas were 0.197 mm^2^ (Fig. 3(A)). Computed tomography revealed a hyperdense plaque predominantly located at the posterior pole of the left eye (Figure 1(i)). The patient was diagnosed with decalcified CO with CNV and SRF.

After receiving an appropriately detailed explanation of the procedure, she was treated via intravitreal aflibercept(2.0 mg) injection once. One month after the therapy, the BCVA was improved to 20/30, OCTA and B-scan revealed that the SRF was increased but the flow areas of CNV were narrowed to 0.191 mm^2^ (Fig. 3(B)), however, 2 months follow-up OCTA revealed that the flow areas of CNV were expanded to 0.666 mm^2^ (Fig. 3(C)), the BCVA was dropped to 20/50. At the last follow-up examination, after 12 months, there were no obvious changes in the fundus, and the BCVA remained 20/50 in her left eye.

## 3 Discussion and conclusions

CO predominantly occurs in healthy young female patients, unilaterally,^[1]^ but its etiology remains unclear. Based on coloration distinctions, CO has been reported to involve varying degrees of decalcified and calcified regions. Evidences suggested that CNV was the typical associated complication of CO.^[6]^

Comparing to fundus fluorescein angiography and indocyanine green angiography, OCTA is a noninvasive imaging modality for depth-resolved visualization of retinal vasculature. Firstly, it provides a high-resolution image of the microvasculature of the different layers in the retina and choriocapillaris layer in the foveal areas.^[7]^ Secondly, OCTA is sensitive to observe the morphology and flow areas of CNV without obscured details due to dye leakage, which occurs on dye-based angiography.^[4]^ The data of flow area values are measured according to the flow signals within the selected areas of CNV.^[8]^ Thirdly, OCTA can compare the morphologic changes of CNV before and after the treatment, provide better assessment of the therapeutic effects.^[4]^ These advantages of OCTA make it widely used in the diagnosis and assessment of CNV in clinical practice. In this present case, OCTA detected the CNV grew into the outer retina layer, 2 different morphologic changes of CNV were also detected during the follow-up period.

There are no clinical guidelines for anti-VEGF therapy of CNV secondary to CO. ^[9]^ In the present case, the patient was treated with intravitreal aflibercept injection. We compared the morphologic changes in the out retina layer before and after treatment, 1 month follow-up, the SRF was increased on OCT B-scan, but the flow areas on OCTA were significantly narrower than the initial situation, and the BCVA was improved to 20/30, however, 2 months after injection, the CNV reoccurred, the SRF was decreased, however, the flow areas of CNV were expanded, and the BCVA was decreased. These evidences proved that OCT B-scan may not reflect the true CNV structure, instead, OCTA can detect the structure and flow areas of CNV more accurately, which correspond the visual state. We speculated the reason for the expanded CNV was that the disease itself was currently in progress, and despite intravitreal aflibercept injection was an effective treatment for CO with CNV, but the effects may not last long. Initial 3 monthly injections of intravitreal aflibercept (2.0 mg) followed by as-needed doses were recommended for treatment of CNV.^[5]^

There were several limitations of this study, this observational study had a small sample size, and the follow-up period after treatment was not long, which only last for 2 months, moreover, the selected CNV areas were performed manually, which may cause some errors. Besides, limitations of OCTA include higher requirement of flow velocity, limited field of view, and inability to identify afferent vessels.^[4]^

In conclusion, we described the OCTA findings of aflibercept for the treatment of CNV secondary to a rare decalcified CO. Decalcification can reduce the functionality of the RPE and induce CNV. The OCTA findings provide a better appreciation of evaluating the effects of intravitreal anti-VEGF drugs for CO complicating CNV. Additional case reports are required in order to better understand the OCTA characteristic of CO.

## Acknowledgment

Not applicable.

## Author contributions

All authors read and approved the final manuscript.

**Investigation:** Bang-tao Yao

**Project administration:** Xiao-gui Zhao

**Supervision:** Bei Wang

**Writing – original draft:** Bang-tao Yao, Fei Wang

**Writing – review and editing:** Yu-hua Ding, Gang Liu
